# Chimeric L2-Based Virus-Like Particle (VLP) Vaccines Targeting Cutaneous Human Papillomaviruses (HPV)

**DOI:** 10.1371/journal.pone.0169533

**Published:** 2017-01-05

**Authors:** Bettina Huber, Christina Schellenbacher, Saeed Shafti-Keramat, Christoph Jindra, Neil Christensen, Reinhard Kirnbauer

**Affiliations:** 1 Laboratory of Viral Oncology, Department of Dermatology, Medical University of Vienna, Vienna, Austria; 2 Department of Pathology, Pennsylvania State University College of Medicine, Hershey, Pennsylvania, United States of America; International Centre for Genetic Engineering and Biotechnology, ITALY

## Abstract

Common cutaneous human papillomavirus (HPV) types induce skin warts, whereas species beta HPV are implicated, together with UV-radiation, in the development of non-melanoma skin cancer (NMSC) in immunosuppressed patients. Licensed HPV vaccines contain virus-like particles (VLP) self-assembled from L1 major capsid proteins that provide type-restricted protection against mucosal HPV infections causing cervical and other ano-genital and oro-pharyngeal carcinomas and warts (condylomas), but do not target heterologous HPV. Experimental papillomavirus vaccines have been designed based on L2 minor capsid proteins that contain type-common neutralization epitopes, to broaden protection to heterologous mucosal and cutaneous HPV types. Repetitive display of the HPV16 L2 cross-neutralization epitope RG1 (amino acids (aa) 17–36) on the surface of HPV16 L1 VLP has greatly enhanced immunogenicity of the L2 peptide. To more directly target cutaneous HPV, L1 fusion proteins were designed that incorporate the RG1 homolog of beta HPV17, the beta HPV5 L2 peptide aa53-72, or the common cutaneous HPV4 RG1 homolog, inserted into DE surface loops of HPV1, 5, 16 or 18 L1 VLP scaffolds. Baculovirus expressed chimeric proteins self-assembled into VLP and VLP-raised NZW rabbit immune sera were evaluated by ELISA and L1- and L2-based pseudovirion (PsV) neutralizing assays, including 12 novel beta PsV types. Chimeric VLP displaying the HPV17 RG1 epitope, but not the HPV5L2 aa53-72 epitope, induced cross-neutralizing humoral immune responses to beta HPV. *In vivo* cross-protection was evaluated by passive serum transfer in a murine PsV challenge model. Immune sera to HPV16L1-17RG1 VLP (cross-) protected against beta HPV5/20/24/38/96/16 (but not type 76), while antisera to HPV5L1-17RG1 VLP cross-protected against HPV20/24/96 only, and sera to HPV1L1-4RG1 VLP cross-protected against HPV4 challenge. In conclusion, RG1-based VLP are promising next generation vaccine candidates to target cutaneous HPV infections.

## Introduction

Papillomaviruses (PV) are a diverse group of non-enveloped, double-stranded, species-specific DNA viruses with strict epithelial tissue tropism. More than 200 human PV (HPV) genotypes have been completely sequenced and classified into five genera: alpha, beta, gamma, mu and nu (pave.niaid.nih.gov; hpvcenter.se) [1]. While genus alpha contains HPV types that infect mucosal or cutaneous epithelia, types of the other genera predominantly infect the skin, although site specificity is not complete [2]. Mucosal HPV can be divided into high (hr) or low-risk (lr) types based upon their oncogenic potential. Alpha HPV infections cause ano-genital warts (condylomata acuminata), and persistent infection with hr types may cause cervical cancers (CxCa), most anal cancers, and a subset of vaginal, vulvar, penile, and oro-pharyngeal (tonsil and base of tongue) cancers. About 13 mucosal hr types, in particular HPV16 and 18, account for practically all CxCa cases worldwide, while lr HPV, most often HPV6 and 11, cause benign genital warts and, infrequently, recurrent laryngeal papillomatosis. Common cutaneous types HPV1, 2, 3, 4, 10, 27, 57 induce benign common and palmo-plantar warts, a frequent nuisance in children, but also in adults and immunosuppressed patients, causing a significant burden to health care systems [3]. Most HPV infections and associated lesions eventually regress due to a cell-mediated immune response [4]. However, in immunosuppressed patients including HIV positive and organ transplant recipients (OTR), warts are more numerous, tend to persist, are more difficult to treat and prevalence increases with time of immunosuppression [5–7].

A group of HPV types belonging to genus beta are hypothesized to play a role as adjunct to the main carcinogen UV light in the development of non-melanoma skin cancers (NMSC) in immunosuppressed OTR and HIV-positive patients. Although beta HPV are detected in skin swabs or hair bulbs from normal individuals by highly sensitive nested PCR, infections appear to be contained by the immune system. Beta HPV have originally been identified in patients with the rare genodermatosis Epidermodysplasia verruciformis (EV), who typically present with generalized flat warts and scaly plaques. Early in life EV patients develop multiple NMSC, squamous cell carcinomas (SCC) or basal cell carcinomas (BCC), predominantly on sun-exposed areas [8]. The genodermatosis is most often inherited in an autosomal recessive manner and 90% of patients show homozygous mutations in EVER1 or EVER2 genes that encode for membrane transporters important for zinc homeostasis [9, 10]. This defect appears associated with increased susceptibility for infections exclusively with beta HPV, and especially hr HPV5 and 8 are found in 90% of EV skin cancers. Beta HPV DNA is also detected in approximately 80% of SCC in OTR, who have a 65-100-fold increased risk to develop NMSC compared to the normal population [11].

While the role of beta HPV causing NMSC in EV patients is long established, their role in development of NMSC in OTR is less clear. Beta HPV infections of the skin are acquired very early in life, even a few days after birth, reaching an overall prevalence of approximately 90% with increasing age without causing clinical symptoms. Studies have revealed increased beta HPV DNA carriage in cutaneous SCC compared to perilesional skin, whereas prevalence and viral load appear higher in precancerous actinic keratoses, opposite to the findings in EV patients [12–14]. In contrast to hr mucosal HPV-associated or EV cancers, the beta HPV genome appears never integrated into the host DNA [10]. Studies on the mechanism how beta HPV may cause skin cancer revealed that beta HPV affect cell cycle and regulatory pathways less profoundly than and via mechanisms somewhat distinct to hr mucosal HPV types [15]. Low viral load and lack of E6 and E7 dependence of cancer progression point towards a role of beta HPV in the early stages of skin carcinogenesis, but not in tumor maintenance (i.e. a potential “hit-and-run” mechanism).

Licensed HPV vaccines are based upon recombinant major structural protein L1 self-assembled into VLP that are immunologically and morphologically similar to native virions [16, 17]. The main mechanism of vaccine efficacy is the induction of high-titer neutralizing antibodies that provide type-restricted protection against infections with vaccine-included HPV types. Vaccination is most beneficial prior to onset of sexual activity, whereas there is no therapeutic effect on existing infections or induced lesions. Licensed vaccines target HPV16/18 (bivalent Cervarix^®^), HPV16/18/6/11 (quadrivalent Gardasil^®^), or HPV16/18/31/33/45/52/58/6/11 (nonavalent Gardasil-9), with the potential to protect against 70–90% of all CxCa cases and, for the latter two vaccines against 90% of genital warts. However, they do not protect against the mucosal types associated with the remaining 10% of CxCa cases requiring cervical screening programs to be maintained in vaccinated women [18]. In addition, they are unlikely to target any cutaneous HPV because of the type-restricted nature of the induced neutralizing immune response and limited cross-protection to even very closely related mucosal types.

Immunosuppressed patients could tremendously profit from a vaccine targeting a broader type spectrum including mucosal and cutaneous HPV since they are often infected with unusual and rare types [6, 19, 20]. HPV vaccinations have proven safe and immunogenic in immunosuppressed patients, inducing seroconversion in 95–100% of HIV-positive patients, but antibody titers were lower compared to healthy controls, which might be improved by additional vaccine boosts [21–23]. Importantly, a prevalent infection with one vaccine-targeted HPV type did not negatively influence antibody induction against other vaccine types, and no effect was seen on CD4+ cell counts or HIV RNA levels. Few studies have been conducted in OTR populations, but available data suggests that licensed HPV vaccines are safe, yet of suboptimal immunogenicity when applied post-transplantation [24]. Less than 50% of OTR seroconverted after Gardasil immunization, and patients immunized early after transplantation showed the lowest responses, suggesting that OTR should be ideally vaccinated prior to transplantation.

In order to extend the type-restricted protection provided by current L1-VLP HPV vaccines towards more distantly related types, several groups have employed the minor capsid protein L2 of PV as immunogen, which can induce low-level neutralizing antibodies to the homologous types, and also cross-neutralization to heterologous HPV types [25]. Several type-common epitopes have been identified within the highly conserved N-terminal amino acids (aa) 11–200 of L2. Vaccination with bovine PV type 1 (BPV1) L2 proteins aa1-88 or aa11-200 of [26, 27], or HPV16 L2 synthetic peptides aa56-75, aa69-81, aa108-120, or aa58-64 induced cross-neutralizing immune responses [28, 29]. In particular, an L2 epitope designated ‘RG1’ comprising aa17-36 of HPV16 L2 was identified by a neutralizing mouse monoclonal antibody (mAb), and HPV16 L2 aa17-36 peptide immunizations provided broad cross-neutralizing activity against heterologous (mostly genus alpha) PV types [30, 31].

Although L2 is incorporated into VLP when co-expressed with L1, L2 is immunologically subdominant in vaccinations with L1/L2 VLP or native virions, or in natural infection due to its minor representation compared to L1, and because L2 is mostly hidden below the capsid surface until the capsid binds the cell surface in vitro or basement membrane in vivo and undergoes a conformational change.

Several strategies have been employed to increase the low immunogenicity of L2 peptides [28, 30, 32–34], including concatameric fusion peptides comprised of L2 epitopes of different HPV types [35–37], coupling to immune activating toll-like receptor agonists [32, 38], or using repetitive surface array of L2 epitopes on particulate immunogenic platforms including *Lactobacillus casei* [39], bacteriophages PP7 or MS2 [40–42], VLP of Tobacco mosaic virus, Adeno-associated virus [43, 44], or bacterial thioredoxin [45].

Another promising approach is the surface display of L2 epitopes by VLP assembled from chimeric HPV L1-L2 fusion proteins. The highly conserved HPV16 L2 B cell epitope RG1 [31, 32] was genetically inserted into the DE surface loop of the HPV16 L1 protein resulting in its multivalent (360x) immunogenic presentation on the VLP surface, while the conformational neutralization epitopes of the HPV16 L1 scaffold were largely maintained [46, 47]. Immunizations with these chimeric HPV16L1-16RG1 VLP (RG1-VLP) induced high type-specific titers to HPV16 and broad cross-neutralization to heterologous mucosal and distantly related cutaneous HPV. In a passive transfer mouse model, antisera protected against genital challenge with 21 mucosal HPV pseudovirions (PsV) including 13 high-risk (hr) types. Importantly, *in vivo* vaccine efficacy was long lasting, providing cross-protection against heterologous HPV58 challenge one year after vaccination in mice. Based on these promising findings the US NCI PREVENT Cancer Preclinical Drug Development Program is currently producing clinical grade HPV16L1-16RG1 chimeric VLP vaccine for IND-enabling studies and proof of concept testing in humans [48].

Immunosuppressed patients could significantly benefit from a vaccination strategy targeting a larger spectrum of HPV types, which is unlikely to be achieved by type-restricted L1 VLP vaccines. Encouraged by the broad spectrum protection achieved by our previous RG1-VLP vaccine, the aim of this study was to design L2-based chimeric immunogens that specifically target cutaneous HPV, using L1 VLP of mucosal or cutaneous types as scaffold. Using this strategy, L1 fusion proteins were generated that display the inserted L2 peptide repetitively and closely spaced on the VLP surface which is expected to greatly increase the L2 peptide’s immunogenicity and to retain at least some of the type-specific L1 conformational epitopes required for induction of high-titer neutralizing antibodies. Because of their less complex (single- or oligo-valent) antigen formulation L2-displaying VLP vaccines are potentially less expensive than multivalent L1 VLP vaccines, with the capacity to greatly increase the protective spectrum. Eventually, it is envisaged to combine several chimeric VLP as a pan-HPV vaccine to provide protection against all relevant cutaneous and mucosal HPV types associated with human pathologies.

## Materials and Methods

### Design of chimeric VLP

Genes encoding chimeric fusion proteins were designed using CLC DNA workbench (CLC bio) based on either HPV5 L1/L2 (NC_001531), HPV16 L1 (HPV16 L1 variant 114K; EU118173) and HPV17 L2 (X74469), HPV1 L1 (NC_001356) and HPV4 L2 (NC_001457), or HPV18 L1 (AY262282) GenBank entries. Genes were codon optimized for Spodoptera frugiperda (Sf9) insect cell expression and synthesized by GeneArt^®^ (Thermo Fisher Scientific). The fusion proteins were designed in two different ways; either by insertion of the HPV17 L2 RG1 epitope (aa14-33) into the DE loop of HPV16 L1 at position aa136/137 for HPV16L1-17RG1, or HPV4 L2 RG1 epitope (aa14-33) at position aa127/128 for HPV1L1-4RG1, with one and two alanines as spacers added before and after the epitope, respectively; or using the HPV17 L2 RG1 epitope (aa14-33) to replace HPV5 L1 aa132-145 of the DE surface loop for HPV5L1-17RG1. Similar to these chimeric L1-RG1 proteins, L1-HPV5 L2 fusion proteins were designed by inserting the HPV5 L2 aa53-72 peptide (a homolog of the previously described cross-neutralization epitope HPV16L2 aa58-64 [29]) into the DE loop of HPV16 L1 or HPV18 L1 (at aa position 136/137 or 134/135, respectively). Chimeric genes were synthesized and cloned (Eurofins Genomic AT, Vienna) into the pOET1 vector (Oxford Expression Technologies, Oxford).

### Expression, purification and characterization of VLP

Fusion genes were sub-cloned into the pSynwtVI- baculovirus transfer vector, co-transfected with linearized baculovirus wild type (wt) DNA (BaculoGold; BD Bioscience) into Sf9 cells and recombinant baculoviruses isolated by plaque purification [16]. HPV16L1-5L2(aa53-72) and HPV18L1-5L2(aa53-72)-expressing pOET1 vectors were used to transfect Sf9 cells using the flashBAC^™^ system according to company’s instructions (Oxford Expression Technologies, Oxford).

For recombinant protein expression, Sf9 insect cells were infected with amplified high-titer baculovirus stocks at high multiplicity of infection for three days. Cells were harvested, lysed by sonication and high molecular weight complexes purified by ultracentrifugation on sucrose-PBS cushions [35% (wt/v)] and CsCl-PBS [29% (wt/wt)] density gradients [49].

Viral fusion proteins were identified by SDS-PAGE / Coomassie staining or Western Blot using mAb Camvir-1 (1:10,000 dilution; BD Pharmingen) [50] or a HPV5 L1 VLP-raised serum [51] directed against L1, or a polyclonal anti-L2 serum (both 1:5,000 dilution; kindly provided by Richard Roden, Johns Hopkins Univ., Baltimore). Self-assembly of chimeric proteins into spherical capsids (VLP) of 50-60nm diameter was verified by transmission electron microscopy (TEM) using a JOEL 1010 electron microscope at 80kV. Briefly, purified VLP were adsorbed onto glow-discharged copper grids, fixed with 2.5% glutaraldehyde and negatively stained with 1% uranylacetate. Micrographs were taken at 30,000x magnification.

### Immunizations

Gradient-purified proteins were extensively dialyzed against PBS +0,5M NaCl +1mM CaCl_2_ +0.02% Tween 80. Fifty μg of respective chimeric VLP were adjuvanted with 500μg alum plus 50μg MPL (alum-MPL; Sigma) and used to immunize two NZW (New Zealand White rabbits) (Charles River Laboratories, Germany) each in a 5-dose regimen at week 0-3/4-6-8-16 for HPV1L1-4RG1 VLP, or in a 4-dose regime at week 0-4-6-8 for HPV16L1-17RG1, HPV5L1-17RG1, 16L1-5L2(aa53-72) or 18L1-5L2(aa53-72) VLP, and sera drawn two weeks after the final boost.

### ELISA

Antigenicity of chimeric L1-RG1 or L1-L2(aa53-72) VLP as compared to wt HPV16L1 or wt HPV18L1/L2 VLP was analyzed by ELISA. VLP preparations were quantified by SDS-PAGE Coomassie staining by visually comparing the intensity of the L1 band at ~50 kDa to serial 1:2 dilutions (from 1 mg/ml to 0.125 mg/ml) of a bovine serum albumin standard (possible impurities or degraded L1 proteins were not taken into account). In short, 0.1μg native VLP per well in 100μl PBS were attached onto Maxisorp 96-well plates (Nunc) over night at 4°C. For denaturing conditions, 0.1μg VLP were dried onto the ELISA plate without a lid in 0.2M NaHCO_3_ (pH 10.6) +0.01M freshly added dithiothreitol (DTT) buffer over night at 37°C. The next day wells were washed, blocked with 0.5% milk/PBS, and serial dilutions of antibodies (all ranging from 1:200–1:204,800) added in triplicates for one hour at room temperature (RT). Antibodies used were neutralizing anti HPV16 L1 mouse mAb H16.V5, H16.5A, H16.14J, 263.A2, H16.E70, or neutralizing anti HPV18 L1 mAb H18.J4 (all kind gifts from Neil Christensen, Penn State, Hershey, Pennsylvania), or non-neutralizing mAb Camvir-1 directed to a linear HPV16 L1 epitope (BD Pharmingen), or rabbit serum raised against HPV16 L2 aa11-200 (a gift from Richard BS Roden, Johns Hopkins). Chimeric HPV5L1-17RG1 and HPV1L1-4RG1 VLP were evaluated using the HPV16L2 aa11-200 polyclonal serum as no Ab to HPV1 or HPV5 L1 were available. After a PBS wash step, the second antibody goat anti-mouse or goat anti-rabbit IgG-HRP (Bio-Rad) was added at 1:10,000 dilution for 45 minutes at RT, followed by substrate 2,2'-azino-bis(3-ethylbenzothiazoline-6-sulphonic acid) (Roche). The OD at 405nm was determined with an ELISA reader (Opsys MR, Dynex Technologies) and positive titers are reported for OD values greater than the mean OD of pre-immune sera plus 3 standard deviation (SD).

### L2 peptide ELISA

To evaluate immunogenic display of the genetically inserted L2 epitopes on the VLPs’ surface, peptide ELISAs were performed using biotinylated RG1-homology peptides of either HPV17 or HPV4, or the HPV5 L2 epitope aa53-72 (JPT peptide technologies, Germany). Briefly, 1μg biotinylated peptide in 100μl coating buffer (1M Tris/HCl (pH7.4) +1M NaCl +0.001% Tween-20) per well were plated at 4°C overnight onto Streptavidin plates (Nunc immobilizer Streptavidin F96 clear). Wells were then washed with coating buffer for 5 minutes and blocked with 1% milk/PBS at 4°C overnight, washed again and incubated in triplicates with serial dilutions of antisera (1:200–1:204,800) for one hour at RT on an ELISA plate shaker. Pre-immune or immune sera raised against wt HPV18L1, HPV16L1, HPV5L1 VLP, or a human HPV1-reactive serum were used as controls. The second antibody goat anti-rabbit IgG-HRP (Bio-Rad) was added for 45 minutes at dilution 1:10,000 at RT, prior to adding the substrate 2,2'-azino-bis(3-ethylbenzothiazoline-6-sulphonic acid) (Roche). The OD at 405nm was determined as stated above and titers are reported for values greater than the mean OD of pre-immune sera plus 3 standard deviation (SD).

### Beta HPV Pseudovirions

Altogether 12 new beta HPV PsV, namely HPV15 (GenBank: X74468), HPV17 (X74469; used NCBI entry lacks 12 C-terminal aa SLKKRKRKRKYL of L2 in comparison to the HPV PAVE entry; last accessed 09–2016), HPV20 (U31778), HPV23 (U31781), HPV24 (U31782), HPV36 (U31785), HPV37 (U31786), HPV49 (X74480), HPV80 (Y15176), HPV92 (AF531420), HPV93 (AY382778) and HPV96 (AY382779) were designed according to published instructions [52]. L1 and L2 genes were codon optimized using a provided website/program (https://home.ccr.cancer.gov/Lco/codonmodification.htm), further edited by hand, synthesized (GeneArt^®^, Life Technologies) and sub-cloned into the double expression vector pVitro1-neo-mcs (Invivogen). The BamHI-XbaI flanked L1 was cloned into MCS2 using the BglII-NheI sites, while the BamHI-XbaI flanked L2 gene was inserted into MCS1 using the BamHI-AvrII sites (according to R. Roden [53]).

PsV were produced in 293TT cells as described [54, 55]. Briefly, 13*10^6^ 293TT cells were plated into 175cm^2^ flasks and one day later transfected with 38μg DNA each of HPV L1 and L2 expression and reporter plasmids (Turbofect, ThermoFisher Scientific). On the next day media was changed, and 48 hours after transfection cells were lysed in PBS +9.5mM MgCl_2_ +0.5% volume Triton-X100 +0.25% Ammonium sulfate +0,1% Benzonase (of a 25^U^/_μl_ stock) +0,1% Plasmid Safe. For particle maturation cell lysates were incubated 20–24 hours at 37°C. Finally, PsV were salt extracted and purified by ultracentrifugation on Optiprep step gradients (27%, 33%, 39%).

Plasmids for L1 and L2 of HPV16 and HPV18 were provided by J. Schiller, NCI; for HPV5 and 45 by C. Buck, NCI; for HPV3 and 76 by J. Dillner, Karolinska Institute; for HPV27 and 57 from [56]; and for HPV8, 38, 39, 59, 68, 70 by R. Roden, Johns Hopkins University. Used reporter were pYSEAP (encoding secreted alkaline phosphatase) and pClucf (encoding luciferase; kindly provided by R. Roden).

Expression of L1 and L2 proteins of novel beta PsV was verified by SDS-Coomassie staining and Western Blot using either mAb Camvir-1 (BD Pharmingen) [50], mAb AU1 (Covance) [57] both at 1:10,000 dilutions, anti-PsV20, anti-PsV38, anti-HPV92L1 VLP, or anti-HPV16L2 aa1-88 polyclonal sera (R. Roden, Johns Hopkins University), all at 1:5,000 dilution. Further, second antibodies goat anti-mouse or anti-rabbit IgG-horseradish peroxidase (HRP) (1:20,000 dilution; Bio-Rad) and ECL Western Blotting substrate (Pierce; #32106) were used. Assembly into virions was verified by transmission electron microscopy (TEM) as described before.

In order to generate type-specific control immune sera, one NZW rabbit each was immunized with HPV4, 20, 24, 36, 38, 39, 59, 68, 76 and 96 in a 3-dose regime at week 0-3-6 using 20μg PsV and Freund’s adjuvants (CFA/IFA) and sera finally drawn two weeks after the final boost (Charles River Laboratories). Additionally, HPV15, 23, 37, 49 and 80 PsV were used to immunize groups of mice (n = 5) subcutaneously with 50ng PsV plus alum (Alhydrogel InvivoGen, as per company’s protocol) as adjuvant in a 2-dose regime (week 0–2) and sera were drawn and pooled two week after the final boost.

A gene encoding 17RG1x5 peptide (optimized for bacterial expression) was synthesized (GeneArt^®^, Life Technologies), subcloned into pET28a vector and, and used to transform Rosetta DE3 cells (Novagen). Protein expression was induced by IPTG (isopropyl-β-D-thiogalactopyranoside) as per company’s instructions and purified via its His-tag using NTA columns (Ni-NTA Spin Kit, Qiagen). 300μg peptide adjuvanted with CFA/IFA were used to immunize two NZW in a 3-dose regime at week 0-3-6, and final immune sera drawn two weeks after the final immunization.

### PsV-based neutralization assays (PBNA)

The L1-based assay was performed as previously described [54]. Briefly, 3*10^4^ 293TT cells per well were plated onto a 96-well plate and infected with SEAP-containing PsV that were pre-incubated without or with (pre-) immune sera on ice, for one hour. Infection was assessed three days later by measuring the SEAP signal at 405nm (Opsys MR, Dynex Technologies) after addition of 4-nitrophenyl phosphate disodium salt hexahydrate (Sigma; N2765) dissolved in diethanolamine (Sigma). Additionally, neutralization assays of types that showed low infectivity (e.g HPV15, 23, 36, 37, 49, 80 or 92) were evaluated using Ziva Ultra SEAP Plus Kit (Jaden BioScience) or GreatEscape SEAP chemiluminescence kit (Cat. #631738; Clontech) according to company’s instructions. In short, after 72 hour incubation, plates were shaken for a minute to gain a homogenous supernatant prior centrifugation for 5 minutes at 1700xg. 5 or 20μl were then transferred onto an opaque 96-well Optiplate (Nunc) containing 75μl 1x SEAP Sample Preparation Solution. Plates were shaken again and incubated at 65–68°C for 50 minutes. Following this inactivation of endogenous SEAP, 25μl (for the ZIVA Ultra SEAP Plus Kit) or 100μl (for the GreatEscape SEAP Kit) of substrate were added and plates incubated in the dark for 30 minutes prior reading luminescence for 0.2–0.3 seconds per well using either the Victor3 or Fluoroskan Ascent FL plate reader. Serum dilutions showing at least a 50% reduction in SEAP signal compared to pre-serum were considered to be neutralizing.

The L2-based assay was performed according to Day et al [58]. In short, 2*10^6^ HaCaT cells were plated onto 96-well plates for 24 hours at 37°C, cells were washed twice with PBS and lysed using PBS +0.5% Triton X-100 +20mM NH_4_OH for 5 minutes at 37°C. Following lysis, 100μl PBS were added and removed again for three times to gently wash the remaining extracellular matrix, and PsV in 120^μl^/_well_ CHOΔfurin conditioned medium containing 5^μg^/_ml_ heparin (Gilvasan Pharma) added. The ECM/PsV mix was incubated overnight at 37°C and the next day washed with PBS to remove non-attached PsV. Antibody serial dilutions were added and incubated at 37°C for at least 1 hour. After this incubation step, 8*10^3^ PGSA-745 cells were directly added to the wells (50^μl^/_well_) and incubated at 37° for two days. For luciferase encoding PsV, assays were evaluated using the Luciferase Assay System (Promega #E1501) and the Cell Culture Lysis Reagent (Promega #E153A) according company’s instructions in 96-well opaque Optiplates (Nunc). Luciferase activity was measured using either the 1420 Victor3 Multilabel Counter (PerkinElmer) or the Fluoroskan Ascent FL (Thermo Scientific) plate reader, with 10 second per well reading time. For SEAP encoding PsV, assays were evaluated using the Ziva Ultra SEAP Plus Kit (Jaden BioScience) or GreatEscAPe^™^ SEAP Kit (Cat. #631738; Clontech) as mentioned before. For CHOΔfurin conditioned medium, 1*10^6^ cells were grown in 17ml media and supernatants harvested after incubation at 37°C for 4 days. Serum dilutions showing at least a 50% reduction in SEAP signal compared to pre-serum were considered to be neutralizing. PGSA-745 and CHOΔfurin cells were kindly provided by Patricia Day, NCI.

### *In vivo* vaginal challenges

Six to eight weeks-old female Balb/c were obtained from Charles River Laboratory and the challenge experiments were performed according to Roberts et al. [59]. Briefly, groups of mice were pretreated with 3mg progesterone sub-cutaneously (Depocon; Pfizer) three days ahead of passive immunization (20μl, intra-venously) with pre-immune serum (n = 10), immune sera raised against L1 wt VLP (n = 5), chimeric L1-RG1 VLP (n = 5; serum #2 for HPV5L1-17RG1 and HPV1L1-4RG1, serum #3 for HPV16L1-17RG1), or serum against the respective specific type (n = 5). One day later, 20μl of a PsV-carboxymethylcellulose (CMC, Sigma) mixture (1:1 for mucosal and cutaneous types HPV5 and 20; 2:1 for HPV4) or undiluted PsV (for HPV24, 38, 76 and 96) were vaginally installed using a positive displacement pipette (Gilson). The cervical mucosa was gently disrupted by 20x rotating a cytobrush (Cooper Surgical Company; C0012) and further 20μl PsV or PsV-CMC were applied intravaginally. Three days later, infection was evaluated by intravaginal installment of 20μl luciferin (7.5^mg^/_ml_; Promega; 10 minute exposure) and imaging by the IVIS system (Caliper Life Science). Data were analyzed by the Living image software program by drawing uniform regions of interest (ROI) around the luminescence emitting genital area of each mouse to determine the average radiance within the ROI. A group of mice (n = 5) was challenge with CMC only to determine background radiance. Luciferase activity was measured as p/s/cm2/sr (average radiance).

### *In vivo* cutaneous challenge

The cutaneous challenge was performed according Wu et al. [60]. Briefly, mice were shaven at the belly and immediately thereafter infected with 40μl HPV5 PsV. Three days later, 0.75mg D-luciferin was injected s.c. below the site of infection and bioluminescence measured by the IVIS system three minutes later (10 minute exposure). Luciferase activity was measured as p/s/cm2/sr (average radiance) after background subtraction (n = 5 mice infected with PBS).

### Animal welfare

Animal studies were approved by the ethics committee of the Medical University of Vienna and the Austrian Federal Ministry of Science and Research (BMWF-66.009/0173-1l/3b/2011) and animal care was in accordance to the guidelines of the Institute for Biomedical Research Vienna. The vaginal/cutaneous challenges were performed under Ketaminhydrochlorid/Xylazin anesthesia to minimize suffering. Animals were sacrificed by cervical dislocation.

### Statistics

*In vivo* statistical analysis was performed using Graph Pad Prism to evaluate p-values between indicated groups (unpaired, two-tailed t-test, and Welch’s corrected if groups with unequal sizes were analyzed).

## Results

### Chimeric L1 VLP that display L2 epitopes of cutaneous HPV

Chimeric L1-L2 fusion genes were designed that encode L1 capsid proteins of hr mucosal HPV16 or HPV18, hr cutaneous beta HPV5, or common cutaneous HPV1 L1 as scaffold to present L2 peptides via the DE surface loop. The HPV17 (17RG1) and HPV4 (4RG1) L2 peptides, comprising each N-terminal 20 aa14-33, were chosen based on homology to the highly conserved broad cross-neutralization epitope ‘RG1’ of HPV16 L2 [31]. The HPV5 L2 peptide aa53-72 was chosen by homology to another cross-neutralization epitope aa56-75 of HPV16 L2 [29]. In total five chimeric fusion genes i.e. HPV16L1-17RG1, HPV5L1-17RG1, HPV16L1-5L2(aa53-72), HPV18L1-5L2(aa53-72), and HPV1L1-4RG1 were synthesized and cloned into a baculovirus transfer vector. The HPV16 L1 scaffold has previously proven highly efficient in epitope display, though presenting the RG1 epitope of beta HPV17 by beta HPV5 L1 might be more advantageous in providing high-titer type-specific immunity to HPV5. In addition, because of their proven efficacy for foreign epitope display, HPV16 and 18 L1 were chosen to present the HPV5 L2 epitope aa53-72. Both HPV1 and 4 are difficult to target by heterologous single formulation L1-RG1 antigens because of their distant relatedness, thus HPV1 L1 was used as scaffold to present the HPV4 RG1 epitope homolog (HPV1L1-4RG1).

**Fig 1:**
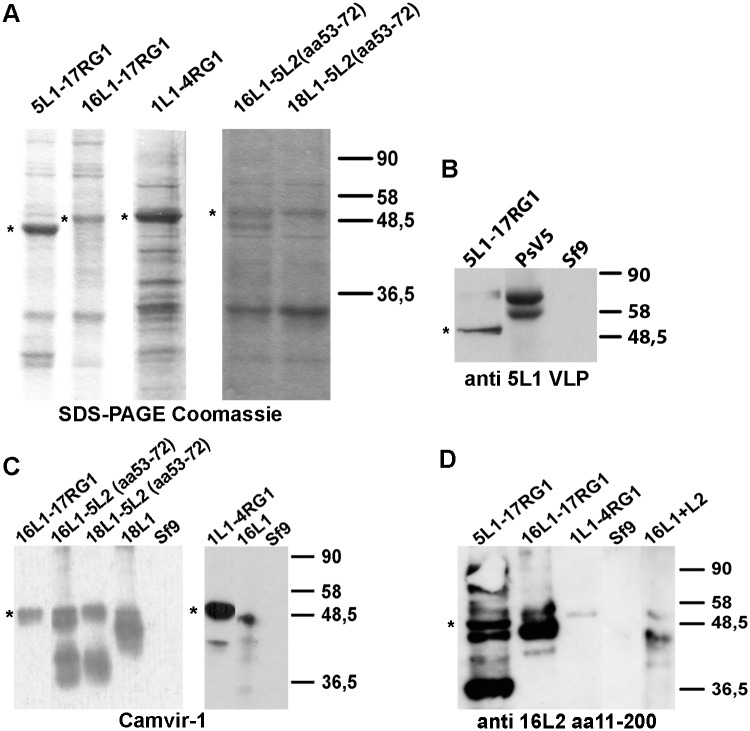
Characterization of chimeric fusion proteins by SDS-PAGE / Coomassie staining and Western Blot. **(A)** VLP purified on density gradients were separated by SDS-PAGE, followed by Coomassie blue staining and verified by Western blot using **(B)** an antiserum raised against HPV5 L1 VLP **(C)** mAb Camvir-1 **(D)** or antiserum raised against HPV16 L2 aa11-200. As indicated, non-infected Sf9 cell extract, HPV5 PsV, wt HPV16L1, HPV16 L1+L2, or HPV18 L1 VLP were used as controls. Recombinant chimeric fusion proteins migrating ~50 kDa are marked with (*).

Recombinant baculoviruses were isolated by plaque purification, amplified, and fusion proteins expressed by infection of Sf9 cells and verified by SDS-PAGE. By Coomassie staining, the five fusion proteins migrated around 50kDa (Fig 1A). Additional bands likely correspond to contaminating Sf9 proteins as well as degradation products and oligomers of recombinant L1-RG1 proteins. By Western Blot, anti-L1 mAb Camvir-1 (Fig 1C) recognized HPV16L1-17RG1, HPV16L1-5L2(aa53-72), HPV18L1-5L2(aa53-72) and HPV1L1-4RG1, whereas HPV5L1-17RG1 was identified by an HPV5 L1 VLP-raised serum (Fig 1B). The respective L2 peptides in fusion proteins HPV5L1-17RG1, HPV16L1-17RG1 and HPV1L1-4RG1 were verified by an antiserum raised against HPV16 L2 aa11-200 (Fig 1D). Additionally, larger bands corresponding to multimers, and smaller bandings indicating degradation products can be observed that appear specific for chimeric proteins, as the uninfected Sf9 cell extract did not show any reactivity with used mAb and polyclonal serum (Fig 1B, 1C and 1D). As indicated, wt HPV16L1, HPV16 L1+L2, or HPV18 L1 proteins were used as controls. Unexpectedly, the antiserum did not recognize the HPV5 L2 epitope aa53-72 of constructs HPV16L1-5L2(aa53-72) and HPV18L1-5L2(aa53-72) (not shown). Information on all mAb and polyclonal sera used regarding specificity and (if known) epitopes is listed in S1 Table.

**Fig 2:**
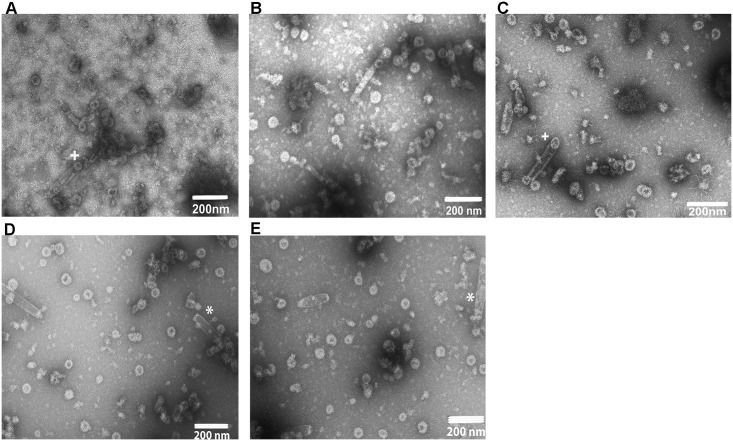
Transmission electron microscopy images of chimeric VLP. Purified preparations of HPV5L1-17RG1 **(A)**, HPV16L1-17RG1 **(B)**, HPV1L1-4RG1 **(C)**, HPV16L1-5L2(aa53-72) **(D)** and HPV18L1-5L2(aa53-72) **(E)** were negatively stained and visualized at 30,000-fold magnification. The size bars represent 200nm. +, tubular structures composed of pentamers; *, co-purified baculovirus contaminant.

Large molecular weight complexes were purified from infected Sf-9 cell extracts on density gradients, negatively stained by uranyl acetate and analysed by transmission electron microscopy (TEM). Electron micrographs showed spherical particles around 50-60nm for chimeric HPV5L1-17RG1 (A), HPV16L1-17RG1 (B), HPV1L1-4RG1 (C), HPV16L1-5L2(aa53-72) (D) and HPV18L1-5L2(aa53-72) (E) fusion proteins (Fig 2), indicating that the chimeric proteins retained the ability to assemble into VLP structures. In addition, tubular structures, aggregates, pentamers, and occasionally co-purified baculovirus were detected.

**Fig 3:**
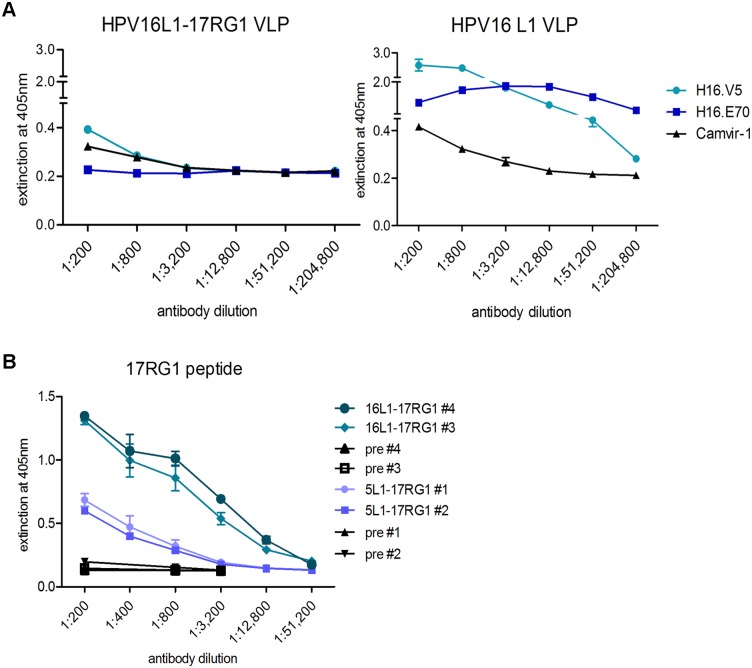
Characterization of chimeric HPV16L1-17RG1 and 5L1-17RG1 VLP by ELISA. **(A)** HPV16L1-17RG1 VLP were compared to wt HPV16 L1 VLP under native conditions using neutralizing mAb H16.V5 and H16.E70, and non-neutralizing mAb Camvir-1. **(B)** Serial dilutions of antisera raised against HPV16L1-17RG1 or HPV5L1-17RG1 were analyzed in triplicates by HPV17RG1 peptide ELISA. Serum titers are reported for OD values greater than the mean OD of pre-immune sera plus 3 standard deviations (SD).

**Fig 4:**
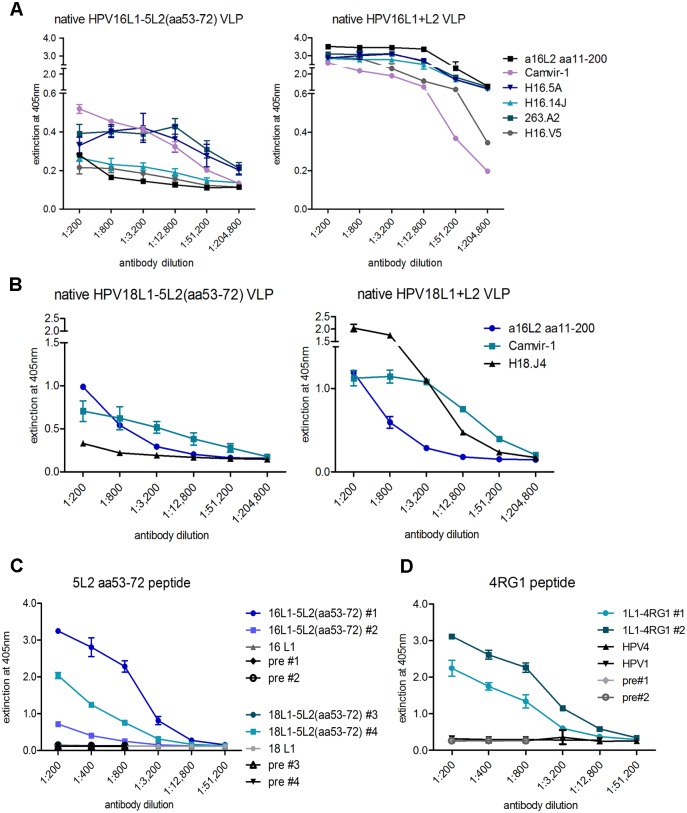
Evaluation of antigenicity and L2 epitope presentation of L1-5L2(aa53-72) and HPV1L1-4RG1 VLP by ELISA. **(A)** Native HPV16L1-5L2(aa53-72) VLP were compared to native wt HPV16 L1+L2 VLP using an antiserum to HPV16 L2 aa11-200, non-neutralizing mAb Camvir-1 and four indicated neutralizing mAb. **(B)** HPV18L1-5L2(aa53-72) VLP were compared to wt HPV18 L1+L2 VLP under native conditions using the anti-L2 serum, Camvir-1 and neutralizing mAb H18.J4. **(C)** Immunogenicity of L2 epitopes presented by either 5L2 aa53-72-based chimeric VLP and **(D)** HPV1L1-4RG1 VLP were evaluated by indicated L2 peptide ELISA using antisera raised against the respective chimeric VLP. Serum titers were graded positive for mean OD values greater than OD of pre-immune sera + 3 SD.

Chimeric VLP were further analysed by ELISA for antigenicity of the VLP scaffold and ability to present inserted L2 epitopes. Under native conditions, HPV16-neutralizing mAb H16.E70 did not bind and H16.V5 only weakly bound to chimeric HPV16L1-17RG1VLP, whereas both mAbs reacted with wt HPV16 L1 VLP as expected (Fig 3A, compare left and right), indicating complete or partial interruption, or steric hindrance of the respective conformational epitopes by L2 peptide insertion into the DE loop. Similarly, weak reactivity with native chimeric VLP was seen using additional neutralizing mAbs H16.5A, H16.14J and 263.A2 (S1A Fig left), which strongly bound to wt HPV16 L1 VLP (not shown) and HPV16L1+L2 VLP (Fig 4A, right), indicating that these mAbs and H16.V5 recognize similar if not identical epitopes. Contrary to non-neutralizing mAb Camvir-1, the neutralizing mAb 263.A2 did not react with HPV16L1-17RG1 VLP under denaturing conditions (S1A Fig, denatured), in agreement with the reported conformational dependence of PV neutralization epitopes. The polyclonal serum raised against HPV16 L2 aa11-200 also reacted with chimeric VLP preferentially under native conditions, while mAb Camvir-1, recognizing a linear epitope, showed stronger binding to denatured VLP (S1B Fig). A similar binding pattern of the anti-L2 serum was seen using HPV5L1-17RG1 VLP, indicating that both beta HPV-targeting chimeric VLP presented the inserted RG1 peptide on the surface with preferential access to Ab (S1C Fig). To evaluate the immunogenicity of chimeric VLP, two NZW rabbits each were immunized 4 times with the respective alum-MPL adjuvanted chimeric VLP and sera were drawn two weeks after the final boost. Analysis of immune sera by a 17RG1-peptide ELISA demonstrated the induction of a humoral response directed against the VLP-inserted HPV17 RG1 peptide (Fig 3B). Of note, HPV16L1-17RG1 VLP-raised L2-peptide titers were higher (12,800 and 51,200 for serum #4 and #3, respectively) compared to those seen for HPV5L1-17RG1 VLP (titers of 800 for both immune sera #1 and #2), indicating that the HPV16 L1 displayed the L2 epitope immunologically more favourably than HPV5 L1.

Next, HPV16L1-5L2(aa53-72) VLP were examined by ELISA and compared to wt HPV16 L1+L2 VLP using conformation-dependent HPV16-neutralizing mAb. Similar to above results, neutralizing mAb binding to native chimeric VLP was weak as compared to wt HPV16 L1+L2 VLP, indicating that epitope insertion interfered with mAb binding (Fig 4A). As expected, binding of the conformation-dependent mAbs was abrogated under denatured conditions (S2A Fig). The HPV16 L2 aa11-200-raised serum very weakly cross-reacted with chimeric VLP containing the HPV5 L2 peptide under both conditions, and control mAb Camvir-1 preferentially bound denatured VLP. Further, HPV18L1-5L2(aa53-72) VLP and wt HPV18 L1+L2 VLP were probed with the HPV18-neutralizing mAb H18.J4 [61]. By ELISA, H18.J4 bound to native wt VLP, but not chimeric VLP, suggesting that peptide insertion interfered with mAb binding analogous to above HPV16 L1 scaffold VLP (Fig 4B). As expected, Camvir-1 predominantly bound to denatured chimeric VLP (S2B Fig). The polyclonal anti-HPV16 L2 aa11-200 serum reacted similarly with both chimeric HPV18 and wt HPV18 L1+L2 VLP. To evaluate immunogenicity, two NZW rabbits were immunized 4 times by either L1-5L2(aa53-72) VLP plus alum-MPL adjuvant and immune sera analysed by HPV5 L2 peptide ELISA. Results indicated immunogenic epitope presentation by chimeric VLP with induced antibody titers of 12,800 and 800 for HPV16L1-5L2(aa53-72) VLP, and a titer of 12,800 for HPV18L1-5L2(aa53-72) VLP-raised serum #4 (Fig 4C).

For chimeric HPV1L1-4RG1 VLP mAb Camvir-1 showed a similar binding pattern by ELISA as seen for the other chimeric VLP, with stronger reactivity to denatured than native VLP (S2C Fig), whereas the HPV16 L2 aa11-200-raised serum cross-reacted to chimeric VLP similarly under both native and denatured conditions. Finally, two NZW rabbits were immunized with HPV1L1-4RG1 VLP adjuvanted with alum-MPL in a 5-dose regime. Immune sera were analysed by L2 peptide ELISA revealing robust induction of antibodies directed against the inserted epitope with titers of 12,800 and 51,200, respectively (Fig 4D).

### Novel beta PsV

**Fig 5:**
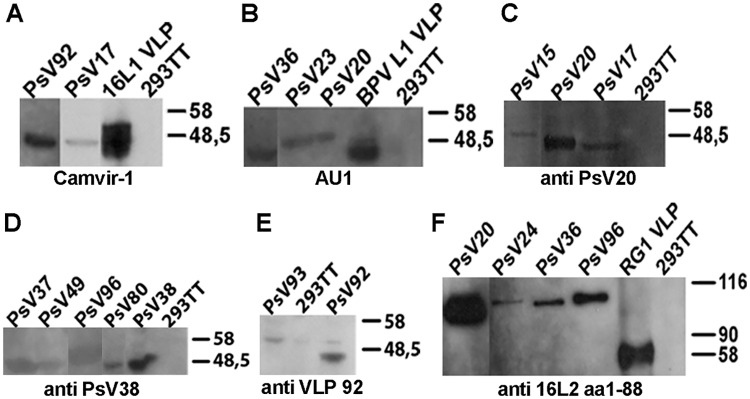
Characterization of L1 and L2 proteins of novel beta PsV by Western Blot. Pseudovirions were generated in 293TT cells and purified preparations analysed by SDS-PAGE and Western Blot. **(A)** MAb Camvir-1, **(B)** mAb AU1, **(C)** or polyclonal antisera raised against PsV20, **(D)** PsV38, **(E)** HPV92 L1 VLP or **(F)** HPV16 L2 aa1-88 were used to detect respective L1 or L2 proteins as indicated. HPV16L1 VLP, BPV1 L1 VLP, RG1 VLP and 293TT producer cells were used as controls.

To generate the tools for evaluating the potential of chimeric VLP immunogens to protect against beta HPV infections, expression vectors for 12 beta HPV PsV types were generated using respective codon-optimized L1 and L2 genes. These comprised HPV15, 17, 20, 23, 24, 36, 37, 49, 80, 92, 93 and 96, representing beta HPV species 1–5 and included types identified in skin cancer samples. Following transfection into 293TT cells and PsV purification on density gradients, L1 plus L2 capsid proteins were verified by Western Blot using mAb to L1, or polyclonal sera raised against heterologous beta PsV types if mAb were non-reactive. MAb Camvir-1 recognized HPV92 and HPV17 L1 proteins (and HPV16 L1 VLP used as positive control) (Fig 5A), whereas mAb AU1 identified HPV36, 23 and 20 L1 (and BPV1 L1 positive control) (Fig 5B). A polyclonal rabbit serum raised against HPV20 PsV further reacted with L1 of HPV15, 17 PsV (and HPV20 PsV as control) (Fig 5C), while antiserum to HPV38 PsV recognized L1 of HPV37, 49, 96 and HPV80 PsV (and of homologous HPV38 PsV as control) (Fig 5D). HPV92 and 93 PsV reacted with a serum raised against HPV92 L1 VLP (Fig 5E). The polyclonal serum to HPV16L2 aa1-88 verified L2 expression in preparations of beta HPV PsV types HPV20, 24, 36 and 96 PsV (Fig 5F) as bands migrating around 100kDa. A fusion protein of HPV16 L1-L2 peptide RG1 (RG1 VLP) migrating around 60kDa served as a control. Other beta HPV PsV did not react with this polyclonal serum, nor with another immune serum raised against HPV16L2 aa1-200, most likely due to low L2 sequence homology. Also antiserum to 17RG1x5 peptide reacted with HPV17 and 37 PsV only, while mAb RG1 failed to detect L2 in several PsV preparations (S2 Table).

**Fig 6:**
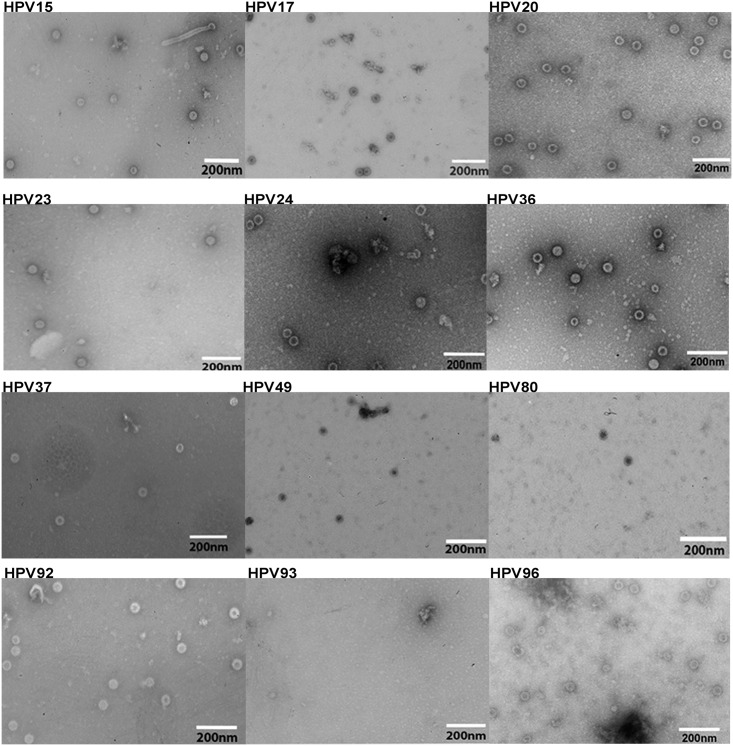
Novel beta pseudovirions visualized by transmission electron microscopy. Purified PsV preparations of indicated HPV types were negatively stained and visualized at 25,000-fold (for PsV 15 and 17), or 30,000-fold magnification by electron microscopy. Size bars indicate 200nm.

Further, assembly of beta HPV L1+L2 proteins into capsids was assessed by TEM of negatively stained PsV preparations. While spherical structures were observed for HPV15, 17, 20, 23, 24, 36, 37, 49, 80, 92 and 96, no capsids were visible for HPV93 PsV preparations (Fig 6). Of note, particles of HPV49 or 80 appeared smaller when compared to other types e.g. HPV20 PsV.

Novel beta HPV were eventually evaluated by *in vitro* neutralization assays, and those with high *in vitro* infectivity were tested *in vivo* in vaginal mouse challenges. HPV17 and 93 proved to be non-infectious *in vitro* even though HPV17 (but not HPV93) preparations showed numerous and correctly sized virions by TEM (Fig 6).

### Evaluating cross-neutralizing potential of chimeric VLP *in vitro*

To assess the potential of chimeric VLP as immunogens, immune sera raised against HPV16L1-17RG1 or HPV5L1-17RG1 VLP were evaluated by both L1- and L2-based assays for neutralization of a large panel of beta HPV PsV types. As shown in Table 1, L1-PBNA detected HPV16L1-17RG1 VLP-raised titers of 10,000 against the homologous HPV16, as well as cross-neutralizing antibodies against HPV8, 20, 24 (titers ranging from 25–100 for at least one of the two sera), whereas HPV5L1-17RG1 VLP raised titers of 50–100 against HPV5, and cross-neutralization against HPV20 and 24 (titers of 50 and 25 for serum #2). As expected, antisera raised against parental wt HPV5 L1 and HPV16 L1 VLP showed high-titer and type-specific neutralization in the respective homologous L1-PsV-based neutralization assay (PBNA), with a neutralization titer of 10,000 against HPV5 (and 100 against closely related HPV8), and 1,000,000 against HPV16, respectively. The lower type-specific antibody titers to HPV15, 37, 49 and 80 likely result from a less rigorous immunization regime and different adjuvant used (titers of 50–100). The more sensitive L2-based PBNA detected improved cross-neutralizing antibody titers (Table 1, shown in bold) induced by HPV5L1-17RG1 VLP vaccination against HPV20, 24, 36, 92 and 96 (titers range from 25–100 for serum #2 only), or by HPV16L1-17RG1 VLP-vaccination that cross-neutralized HPV5, 8, 20, 23, 24, 36, 49, 80, 92 and 96 (titers ranging from 25–1,000).

**Table 1:** Vaccinations with chimeric HPV5L1-17RG1 or HPV16L1-17RG1 VLP induce antisera that cross-neutralize beta HPV types.

L1-based PsV neutralization assay									L2-based PsV neutralization assay						
HPV type/species		anti HPV5L1-17RG1 VLP		anti HPV5 L1 VLP	anti HPV16L1-17RG1 VLP		anti HPV 16L1 VLP	type-specific control	anti HPV5L1-17RG1 VLP		anti HPV5 L1 VLP	anti HPV16L1-17RG1 VLP		anti HPV 16L1 VLP	type-specific control
		#1	#2		#3	#4			#1	#2		#3	#4		
5	β1	50	100	10,000	-	-	-	**10,000**	50	100	10,000	**100**	**100**	-	**10,000**
8		-	-	100	25	50–100	-	10,000	-	-	-	**50**	**100**	-	10,000
20		-	50	-	-	50–100	-	100,000	-	**100**	-	**100**	**100–1,000**	-	100,000
24		-	25	-	-	25	-	10,000	-	**100**	-	**100**	**100**	-	10,000
36		-	-	-	-	-	-	10,000	-	**100**	-	**100**	**100**	-	10,000
15	β2	-	-	-	-	-	-	100	-	-	-	-	-	-	100
23		-	-	-	-	-	-	**-**	-	-	-	**25–50**	-	-	-
37		-	-	-	-	-	-	100	-	-	-	-	-	-	100
38		-	-	-	-	-	-	1,000,000	-	-	-	-	-	-	1,000,000
80		-	-	-	-	-	-	100	-	-	-	**>25**	-	-	100
49	β3	-	-	-	-	-	-	50	-	-	-	-	**>25**	-	50
76		-	-	-	-	-	-	100,000	-	-	-	-	-	-	100,000
92	β4	-	-	-	-	-	-	-	-	**100**	-	**100**	-	-	-
96	β5	-	-	-	-	-	-	10,000	-	**25–50**	-	**25**	-	-	10,000
16	α9	n.d.			10,000	10,000	1,000,000		n.d.			1,000	1,000	1,000,000	

Two NZW rabbits each were vaccinated with chimeric HPV5L1-17RG1 or HPV16L1-17RG1 VLP and immune sera were tested against a panel of different beta HPV PsV for cross-neutralizing activity by L1-based and L2-based PBNA. Improved cross-neutralizing titers detected by L2-based PBNA are shown in bold. Type-specific antisera raised against indicated PsV (type-specific control) or antisera to HPV5 L1 or HPV16 L1 VLP were used as controls. Neutralization titers are indicated for reciprocal serum dilutions resulting in 50% reduction in reporter signal compared to pre-immune serum. Titers of <25 were considered non-neutralizing and indicated as (-). n.d. indicates not determined.

For immune sera raised against HPV16L1-5L2(aa53-72) and HPV18L1-5L2(aa53-72), high neutralization titers were detected against homologous L1-scaffold HPV16 (titers of 1,000–10,000 for sera #1 and #2) and HPV18 (titers of 100,000 for sera #3 and #4) by L1-PBNA (Table 2, left panel). Despite the induction of antibodies to the inserted HPV5 L2 aa53-72 peptide as judged by ELISA (Fig 4C), no (cross-)neutralizing activity was detected against any of 6 beta HPV types (HPV5, 8, 20, 24, 38 and 76) tested, including homologous HPV5 by both L1-PBNA and L2-PBNA (Table 2, right panel).

**Table 2:** Evaluation of HPV16L1-5L2(aa53-72) and HPV18L1-5L2(aa53-72)-mediated immune response by L1- and L2-based PBNA.

L1-based PBNA								L2-based PBNA					
HPV type	genus	anti HPV16L1-5L2 (aa53-72) VLP sera		anti HPV18L1-5L2(aa53-72) VLP sera		anti HPV16+18 L1 VLP	type specific serum	anti HPV16L1-5L2(aa53-72) VLP sera		anti HPV18L1-5L2(aa53-72) VLP sera		anti HPV16+18 L1 VLP	type specific serum
		#1	#2	#3	#4			#1	#2	#3	#4		
5, 8, 20, 24, 38, 76	β	-	-	-	-	-/-	10,000–100,000	-	-	-	-	-/-	10,000–100,000
16	α	1,000	10,000	-	-	10,000/-		n.d.	n.d.	n.d.	n.d.	n.d.	n.d.
18		n.d.	n.d.	100,000	100,000	-/1,000,000		n.d.	n.d.	n.d.	n.d.	n.d.	n.d.

Immune sera raised against chimeric HPV16L1-5L2(aa53-72) VLP or HPV18L1-5L2(aa53-72) VLP were tested for neutralization of indicated beta HPV PsV, or the respective homologous alpha HPV16 and HPV18 PsV. Type-specific sera raised against the respective PsV or VLP were used as controls. Neutralization titers are indicated for reciprocal serum dilutions showing 50% reduction in reporter signal compared to pre-immune serum. n.d. not determined, (-) indicates titers of <25.

Finally, HPV1L1-4RG1 VLP-raised immune sera were analysed by PBNA against a panel of cutaneous HPV types belonging to different genera, including gamma type HPV4, beta types HPV5, 8, 20, 24, 36, 38, 76 and 96, and the cutaneous alpha types HPV3, 27 and 57. Neutralizing antibodies (titers of 100 and 1,000 sera #1 and #2, respectively) were detected by L2-PBNA against HPV4 only (a PBNA for HPV1 is not available), but not against any of the tested common cutaneous (HPV3, 27 and 57) or beta HPV types tested (HPV5, 8, 20, 24, 36, 38, 76 and 96) by L1-PBNA (S5 Table).

### *In vivo* murine vaginal challenges

Following *in vitro* analysis for cross-neutralization, one antiserum each to HPV5L1-17RG1 (serum #2), HPV16L1-17RG1 (serum #3) and HPV1L1-4RG1 VLP (serum #2) was further evaluated for *in vivo* cross-protection using the murine vaginal challenge model [59].

**Fig 7:**
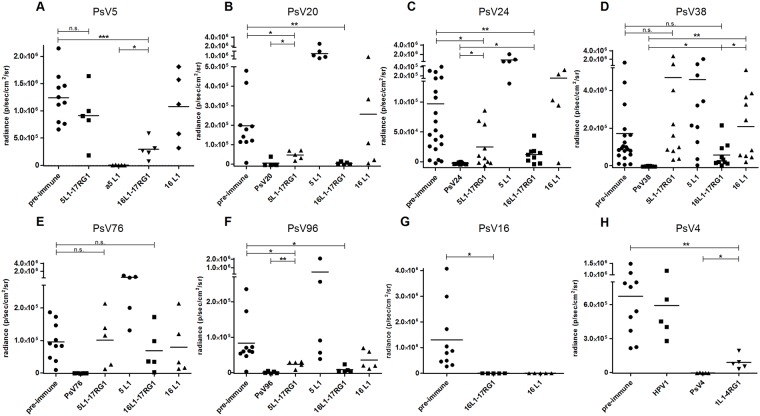
*In vivo* cross-protection conferred by passive transfer of immune sera raised against L1-RG1 VLP in the murine vaginal challenge model. Progesterone pre-treated groups of mice were passively immunized with either pooled pre-immune sera (n = 10), or sera raised against indicated chimeric VLP, HPV5 or 16 L1 VLP (n = 5 each), or sera raised against the respective PsV or VLP (n = 5) as a type-specific controls. As indicated, mice transferred with immune sera to HPV5L1-17RG1 and HPV16L1-17RG1 VLP were challenged with HPV5 **(A)**, 20 **(B)**, 24 **(C)**, 38 **(D)**, 76 **(E)** and 96 **(F)** PsV, whereas the latter serum additionally was evaluated by HPV16 challenge **(G)**. HPV1L1-4RG1 VLP-raised sera were tested for efficacy against HPV4 PsV challenge **(H)**. A human immune serum reactive against HPV1 was used as a control. Luciferase activity was measured as p/s/cm2/sr (average radiance) and quantified after background subtraction (n = 5 mice challenged with CMC only). *, ** and *** indicate t-test p-values of <0.05, <0.005 and <0.0005.

Passive transfer of HPV5L1-17RG1 VLP-raised serum protected mice partially from experimental challenge with HPV20, 24 and 96 (Fig 7B, 7C and 7F, t-test p-values comparing pre-immune and chimeric VLP groups of 0.01, 0.01 and 0.02) but surprisingly not against the homologous type HPV5 (Fig 7A, p-value of 0.27), even though low-titer neutralizing antibodies were detected by PBNA (Table 1, titer of 50–100). The same serum proved to be non-protective against HPV38 and 76 as well (Fig 7D and 7E, p-values of 0.3 and 0.9, respectively).

The HPV16L1-17RG1 VLP-raised immune serum conferred complete protection against challenge with HPV20, 96 and the homologous type HPV16 (Fig 7B, 7F and 7G, t-test p-values comparing pre-immune and chimeric VLP groups of 0.002, 0.007 and 0.01, respectively), as well as partial protection against infection with HPV5, 24 and 38 (Fig 7A, 7C and 7D, p-values of 0.0001, 0.003 and 0.08), but not against HPV76 (Fig 7E, p-value of 0.46).

When mice were challenged with HPV24 and 38 PsV, infectivity was rather low and variable especially in the pre-immune serum group, and hence experiments were performed twice and results pooled (Fig 7C and 7D). Concerning the HPV38 challenge, no significant difference was seen between the pre-immune serum-immunized and any of the chimeric VLP-sera immunized groups (p-value of 0.3 for HPV5L1-17RG1 and 0.08 for HPV16L1-17RG1). At least for the HPV16L1-17RG1 VLP-immunized group significant differences to the anti-HPV16 L1 control, as well as to the anti-HPV38 control were observed, indicating partial protection (p-values of 0.02 and 0.01).

In general, induction of partial protection was reported if there was a significant difference between chimeric VLP group and both pre-immune and type-specific control groups. With the exception of the reported partial protection against HPV38, partial protection was in all other cases in agreement with *in vitro* cross-neutralization (Table 1). This includes partial protection against HPV20, 24 and 96 by HPV5L1-17RG1 VLP (t-test p-values comparing chimeric VLP and type-specific control groups of 0.01, 0.02 and 0.0008, respectively) and corresponding cross-neutralizing titers of 100 and 25–50, respectively, as well as partial protection conferred by HPV16L1-17RG1 VLP-raised serum against HPV5 and 24 (t-test p-values comparing chimeric VLP and type-specific control groups of 0.008 and 0.01, respectively) and corresponding titers of 100 for both types.

For sera induced by HPV1L1-4RG1 VLP vaccination, *in vitro* neutralization assays failed to reveal induction of cross-neutralizing antibody titers (at a minimum dilution of 1:25) against any of the tested cutaneous HPV except HPV4, and thus *in vivo* challenge was performed with this type only. As can be seen in Fig 7H, passive immunization of mice using an HPV1L1-4RG1-raised immune serum partially protected mice against HPV4 challenge as a significant difference of the chimeric VLP to the type-specific group was observed (p<0.05).

## Discussion

While the involvement of beta HPV in NMSC carcinogenesis in immunosuppressed patients is supported by epidemiological and experimental data, a possible role in keratinocyte skin cancer of the general population is less clear. Beta HPV DNA is more frequently detected in premalignant actinic keratosis than in NMSC and only a minority of cancer cells harbor viral DNA, supporting a possible role in initiation but not maintenance of the tumor phenotype [14].

In a recent study using the natural Mastomys natalensis papillomavirus (MnPV) mouse model, prophylactic vaccination of naïve mice, and even therapeutic vaccination of previously infected animals protected against development of MnPV-associated skin cancer [62]. Therapeutic efficacy in infected mice was mediated by neutralizing antibodies that prevented the establishment of novel MnPV-induced skin lesions, although MnPV L1-VLP vaccination failed to eradicate the virus. In humans, natural HPV16 infection induced limited B cell memory and usually non-neutralizing antibodies, whereas a single vaccine dose in HPV16 seropositives induced neutralizing antibodies and memory B cells [63]. Immunization of EV patients and OTR with a vaccine that targets oncogenic beta HPV infections could potentially prevent development of NMSC if administered early. This approach might confirm or refute the hypothesis that beta-HPV play an adjunct role in NMSC, as hit-and-run type mechanisms are very difficult to demonstrate otherwise. Seropositivity for multiple beta HPV types has been linked with increased risk to NMSC development. Organ transplant patients are frequently infected with multiple beta types, and the number of different types increased with duration on immunosuppressive therapy. Thus timely immunization, preferably before start of immunosuppression, might prevent infection with not yet encountered beta HPV types reducing the risk for NMSC development [64].

We have used the RG1 homologs of beta HPV or a common cutaneous type as type-common experimental vaccine antigens to more directly target cutaneous HPV, compared to a previous HPV16-based chimeric RG1-VLP (HPV16L1-16RG1) immunogen [46, 47]. The HPV17 RG1 epitope was chosen because of conserved sequence homology (85–100%) to a panel of different beta HPV raising the possibility to induce broad cross-neutralization within this group (S3 Table).

Previously, expression of recombinant HPV L1 proteins in insect cells has shown that truncation of the nuclear targeting and DNA binding signals at the C-terminus of HPV16 and HPV18 L1 reduced incorporation of cellular DNA, whereas the capacity to assemble in immunogenic VLP was retained [65]. In addition to full length recombinant proteins, smaller bands are observed by SDS-PAGE or Western blot in our chimeric VLP preparations that likely correspond to proteolytic degradation products including C-terminal truncated L1 proteins, i.e. prominent ~45 kDa bands for HPV16L1-5L2(aa53-72) and HPV18L1-5L2(aa53-72), or ~50 kD bands for HPV5L1-17-RG1. We may speculate that chimeric L1 proteins with at least smaller C-terminal proteolytic truncations are incorporated into assembled VLP.

The current standard approach to assess neutralizing antibodies, which are the main effectors of protection induced by L1 VLP vaccination, is by ‘L1- sensitive’ PsV-based neutralization assays (L1-PBNA). However, the L1-PBNA was shown to be less sensitive and thereby possibly underestimating the extent of L2-mediated cross-protective humoral responses. Consequently, a novel L2-based neutralization assay that detects L2-mediated neutralization (L2-PBNA) with higher sensitivity, as well as L1-targeted neutralization, was employed to analyse the (cross-) neutralization spectrum of chimeric VLP [58].

Unexpectedly, for antisera to HPV5L1-17RG1 VLP low type-specific neutralizing titers to homologous HPV5 were detected *in vitro* which were not protective against HPV5 PsV challenge *in vivo*, while cross-neutralization was observed against additional 5 of 13 beta HPV by L2-PBNA. Nevertheless, mice passively immunized with sera raised against chimeric HPV5L1-17RG1 VLP were partially protected against experimental challenge with HPV20, 24 and 96 PsV, but not against HPV38 and 76. These results suggest that 17RG1 epitope insertion into and sequence replacement of the DE loop disrupted or sterically hindered major HPV5 L1 neutralization epitope(s) exposure in the chimeric HPV5L1-17RG1 VLP. Unfortunately, mAb to analyze chimeric and parental HPV5 VLP for type-specific neutralization epitopes were not available. However, the binding of HPV16-neutralizing mAb H16.V5 was recently mapped to 17 residues found in five intertwined loops of two neighboring L1 molecules, comprising the DE and HI loops of one L1 and the BC, DE and FG loops of an adjacent L1 [66, 67]. Involved DE residues have shown to include aa138/139 as well as aa142/143/144 of the two HPV16 L1 molecules, and corresponding residues would have been replaced by the HPV17 RG1 in chimeric HPV5L1-17RG1 VLP (replacement of aa132-145), therefor disrupting a possible homolog of the major neutralizing epitope. Further, HPV5L1-17RG1 also did not mount cross-neutralizing antibodies against the closely related HPV8 as observed for the HPV5 L1 VLP-raised serum, which is in agreement with an abrogated L1-dependent immune response [51].

Neutralizing mAb H16.V5 has been shown to block 75% of reactive human sera with HPV16 VLP. Although reactivity of H16.V5 with HPV16L1-17RG1 VLP was rather weak, immune sera conferred complete type-specific *in vivo* protection against HPV16 PsV challenge, indicating the chimeric HPV16 VLP retained protective immunogenicity. RG1 epitope insertion at L1 positions 136/137 might have disrupted the DE loop involvement in the H16.V5 recognition site, while residual binding might be due to the remaining FG residues. MAb 263.A2 appears to have a similar binding pattern to that of H16.V5, with FG and HI loop residues recognized by both mAb defining a complex epitope recognized by a majority of human sera in response to natural HPV16 infection [66, 68]. No reactivity was seen between chimeric VLP and HPV16-neutralizing mAb H16.E70 whose recognition site was mapped to the FG and DE loop as well [69, 70]. Of importance, HPV16L1-17RG1 VLP were able to raise antisera with type-specific HPV16 titers of 10,000, and cross-neutralization against 10 of 14 tested beta types HPV5/8/20/24/36/23/80/49/92/96. *In vivo* experiments further confirmed immunogen-mediated (cross-) protection against challenge with HPV20, 96 and the homologous HPV16, while partial protection was observed against HPV5, 24 and 38.

Even though chimeric VLP generated herein were primarily designed to target cutaneous HPV, we decided to evaluate *in vivo* protection by murine vaginal challenge, because infection at the cutaneous site with beta HPV5, a type showing usually high *in vivo* infectivity, was much lower and more variable when compared to infection of the vaginal mucosal and thus did not allow robust analysis of vaccine efficacy (S3 Fig). Of note, although previously considered strictly cutaneous types, beta HPV have been found to frequently infect anal and oral mucosa [71, 72].

Novel beta HPV PsV types were designed according to HPV prevalence in NMSC of both immune-suppressed and immune—competent patients, and to provide at least one type representative of different beta HPV species [73]. In general, novel beta HPV PsV showed variable infectivity and not all were useful *in vivo*. Of 12 novel types, HPV17 and 93 PsV showed no *in vitro* infectivity, while by TEM correctly sized PsV were observed for HPV17, but not for HPV93. Re-evaluation of the used HPV17 L1 and L2 sequences (NCBI entry X74469, last accessed 09–2016) revealed that 12 C-terminal aa of its L2 protein were predicted to be missing (residues 525–536: SLKKRKRKRKYL) by comparison to the HPV17 L2 sequence entry from the HPV PAVE homepage (https://pave.niaid.nih.gov/, last accessed 09–2016). The missing sequence is highly conserved to the C-terminus of HPV16 L2 that contains a nuclear localization signal (aa454-462), a nuclear export signal (aa462-471) and a dynein-binding site (the last C-terminal 40 aa) [74–76]. Mutations in the latter site have shown to greatly reduce infectivity possibly resulting from failed transport along microtubules towards the nucleus. This predicts that the HPV17 L2 homolog to this dynein-binding site is missing and likely explains why HPV17 PsV carrying a C-terminal 12 aa deleted L2 were non-infectious while capsid assembly was retained.

The second L2 epitope used in this study is the HPV5 L2 aa53-72 homolog of HPV16 L2 aa56-75. The latter epitope has originally been described to induce cross-neutralizing antibodies against mucosal hr types [29]. More recently the HPV58 homolog of this epitope was inserted either alone, or together with the HPV31 RG1 epitope into the HPV18 L1 C-terminal arm and DE loop. Resulting chimeric VLP immunogens induced cross-neutralization against several tested HPV, including the beta type HPV5, alpha lr HPV11 and hr HPV33/45/58 [77]. These results obtained with mucosal HPV stand in stark contrast to results herein, demonstrating that antisera raised against the HPV5 L2 aa53-72-augmented chimeric immunogens failed to induce cross-neutralization against any of the beta HPV types tested including homologous type HPV5 despite induction of antibodies to the inserted epitope. The HPV5 peptide homolog to HPV16 L2 aa56-75 was regarded a potentially promising epitope because of a >85% sequence homology to a panel of different beta HPV types (S4 Table), including HPV76 or 38 that were not or only partially cross-neutralized by antisera to HPV17 RG1-containing VLP. We may speculate that structural differences between mucosal and cutaneous beta HPV types exist that account for these discrepant results.

Common cutaneous types HPV1 and HPV4 are more difficult to target by a single formulation immunogen due to their distant relatedness to other types, thus we decided to insert the RG1 epitope of HPV4 into the L1 DE loop of HPV1. The polyclonal anti-HPV16 L2 serum showed similar reactivity with chimeric HPV1L1-4RG1 protein under both native and denatured conditions by ELISA, consistent with exposure of a linear L2 epitope on the VLP surface. This was corroborated by L2 peptide ELISA revealing the induction of high anti-RG1 titers by rabbit vaccination with chimeric HPV1L1-4RG1 VLP, thus pointing towards an immunogenic epitope presentation on the VLP surface. We were unable to evaluate whether induced sera neutralize homologous HPV1, as PsV assays are not available for this type despite multiple attempts (Chris Buck, pers. comm.). Antisera were neutralizing *in vitro* and partially protected *in vivo* against HPV4 challenge, likely by targeting the homologous HPV4 RG1 epitope. However, antisera failed to cross-neutralize distantly related cutaneous alpha and beta HPV by PBNA, which is likely due to ≤50% RG1 sequence homology to tested cutaneous types (S5 Table).

In conclusion, chimeric VLP immunogens that display cross-neutralization L2-RG1 epitopes of cutaneous HPV types are evolving as a promising tool to develop next generation L2-based prophylactic vaccines with broad-spectrum protection against HPV types associated with skin pathologies in immunocompetent and immunosuppressed populations.

## Supporting Information

S1 Fig Evaluation of L1-17RG1 chimeric VLP by ELISA. **(A)** Reactivity of non-neutralizing mAb Camvir-1 and HPV16-neutralizing mAb H16.5A, H16.14J, 263.A2 with native or denatured HPV16L1-17RG1 VLP. Analysis of L2 epitope presentation by HPV16L1-17RG1 VLP **(B)** and HPV5L1-17RG1 VLP **(C)** using Camvir-1 and an antiserum raised to HPV16 L2 aa11-200 under native and denatured conditions.
(TIF)

S2 Fig ELISA reactivity of L1-5L2(aa53-72) VLP under denatured conditions and evaluation of immunogenic RG1 presentation of HPV1L1-4RG1 VLP. **(A)** HPV16L1-5L2(aa53-72) and **(B)** HPV18L1-5L2(aa53-72) VLP were compared to wt L1+L2 VLP under denatured conditions using indicated mAb or polyclonal serum. **(C)** Native or denature HPV1L1-4RG1 VLP were analyzed with Camvir-1 and antiserum to HPV16 L2aa11-200.
(TIF)

S3 Fig Comparison of cutaneous and vaginal challenge in mice using HPV5 PsV. Groups of Balb/c (n = 10) were passively immunized with pre-immune serum and infected with 40μl HPV5 PsV at the skin of the belly or the vaginal mucosa as described in Materials. After subcutaneous or intravaginal addition of D-luciferin, bioluminescence was evaluated by in vivo imaging. Data from the vaginal challenge was taken from Fig 7A. Luciferase activity was measured as p/s/cm2/sr (average radiance) after background subtraction.
(TIF)

S1 Table List of mAb and polyclonal sera and their specificity. (TIF)

S2 Table Western Blot-reactivity of mAb and polyclonal sera with beta HPV PsV. MAb Camvir-1 or AU1, or polyclonal sera raised against the indicated L1 and/or L2 antigens, were used to detect L1 and L2 expression of a panel of beta HPV PsV by Western blot. Results are summarized. (-), (~) and (+) indicate negative, weak, or positive immunoreactivity, respectively. Empty boxes indicate that no experiment was done.
(TIF)

S3 Table Comparison of the HPV17 RG1 epitope against a panel of different Beta HPV. (TIF)

S4 Table Sequence homology of HPV5 L2 aa53-72 compared to homologs of other beta types and mucosal hr type HPV16. (TIF)

S5 Table Sequence comparison of the RG1-homologous peptide of HPV4 against analogous peptides of indicated cutaneous HPV belonging to different genera. (TIF)
