# Supplementary figures and images for: Chimeric L2-Based Virus-Like Particle (VLP) Vaccines Targeting Cutaneous Human Papillomaviruses (HPV)
Source: PLoS One. 2017 Jan 5;12(1):e0169533. doi: 10.1371/journal.pone.0169533 (PMC5215943; doi:10.1371/journal.pone.0169533)

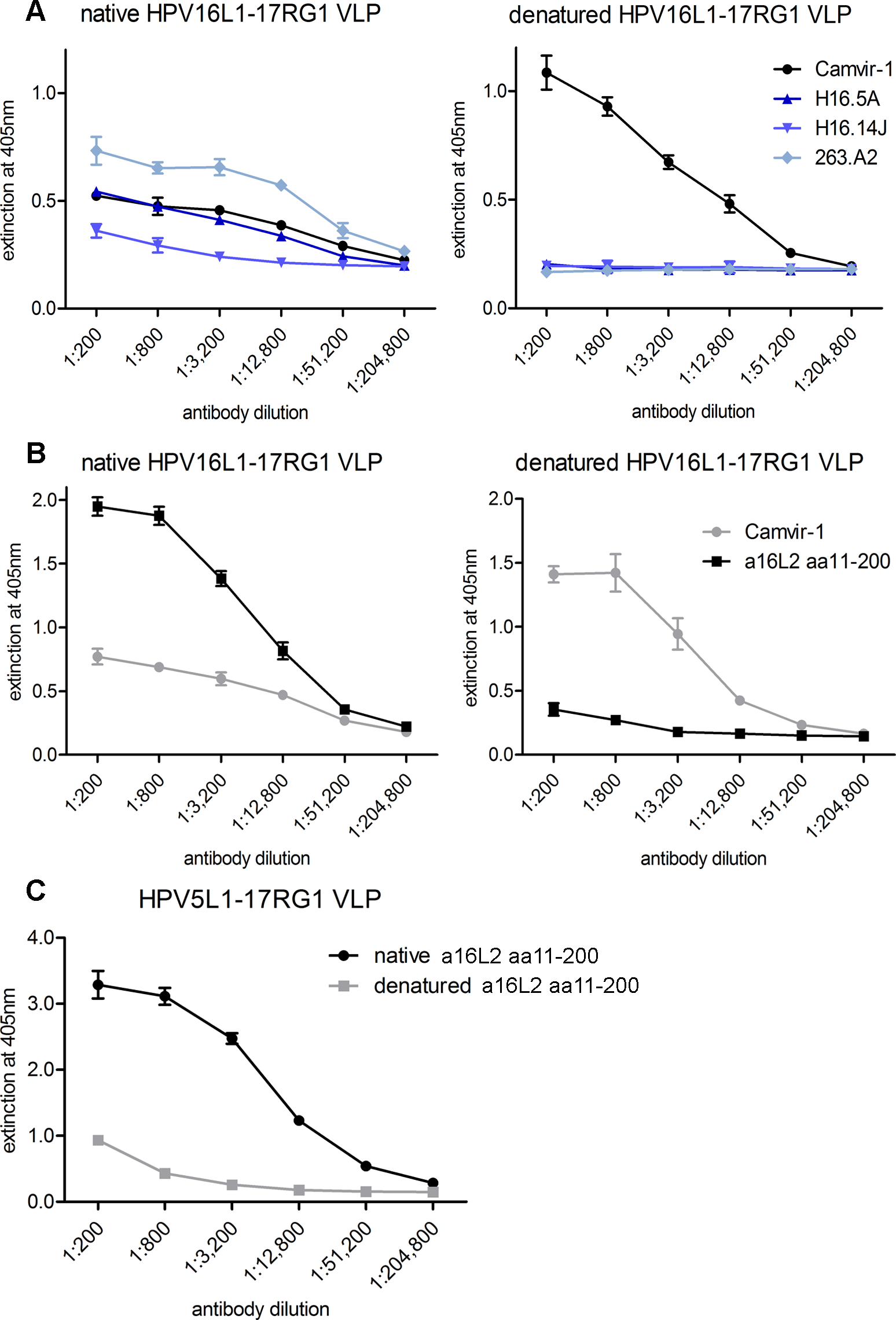

Supplement: S1 Fig — (A) Reactivity of non-neutralizing mAb Camvir-1 and HPV16-neutralizing mAb H16.5A, H16.14J, 263.A2 with native or denatured HPV16L1-17RG1 VLP. Analysis of L2 epitope presentation by HPV16L1-17RG1 VLP (B) and HPV5L1-17RG1 VLP (C) using Camvir-1 and an antiserum raised to HPV16 L2 aa11-200 under native and denatured conditions. (TIF) [file pone.0169533.s001.tif]

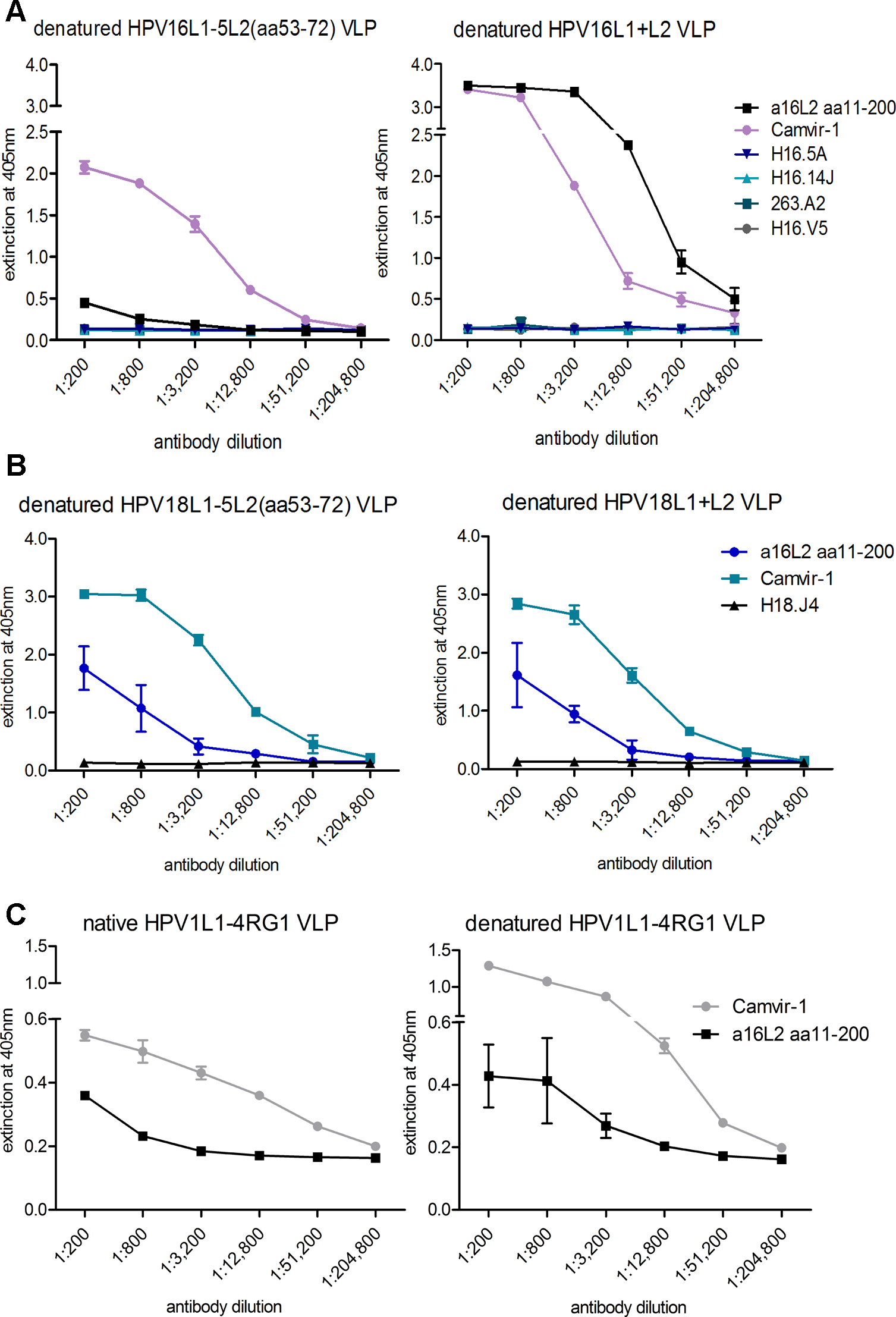

Supplement: S2 Fig — (A) HPV16L1-5L2(aa53-72) and (B) HPV18L1-5L2(aa53-72) VLP were compared to wt L1+L2 VLP under denatured conditions using indicated mAb or polyclonal serum. (C) Native or denature HPV1L1-4RG1 VLP were analyzed with Camvir-1 and antiserum to HPV16 L2aa11-200. (TIF) [file pone.0169533.s002.tif]

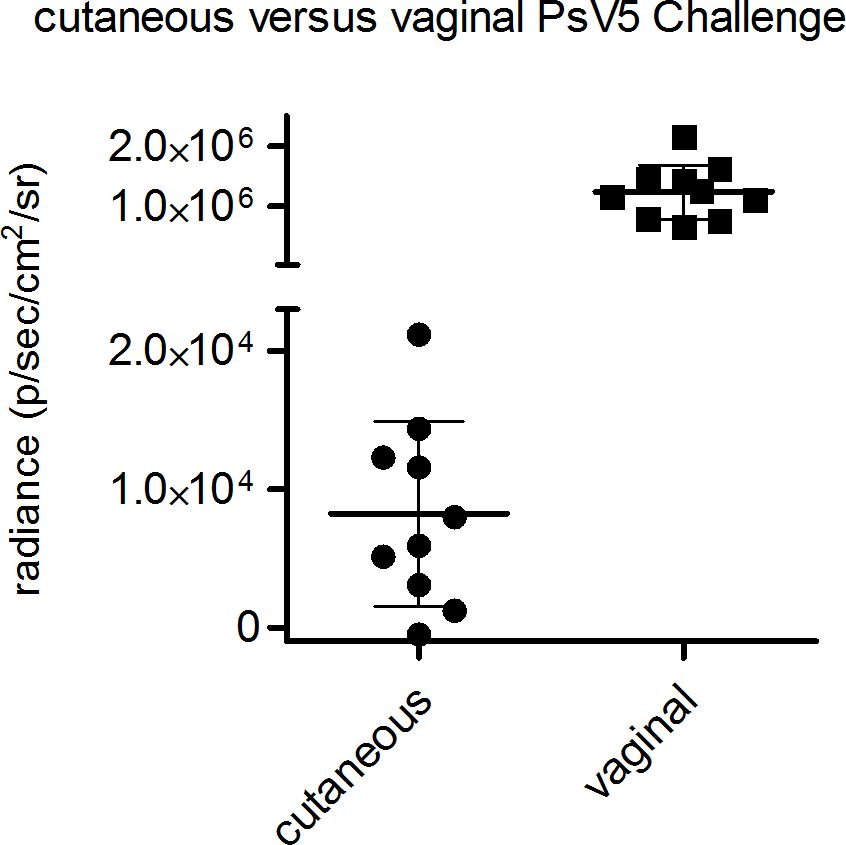

Supplement: S3 Fig — Groups of Balb/c (n = 10) were passively immunized with pre-immune serum and infected with 40μl HPV5 PsV at the skin of the belly or the vaginal mucosa as described in Materials. After subcutaneous or intravaginal addition of D-luciferin, bioluminescence was evaluated by in vivo imaging. Data from the vaginal challenge was taken from Fig 7A. Luciferase activity was measured as p/s/cm2/sr (average radiance) after background subtraction. (TIF) [file pone.0169533.s003.tif]

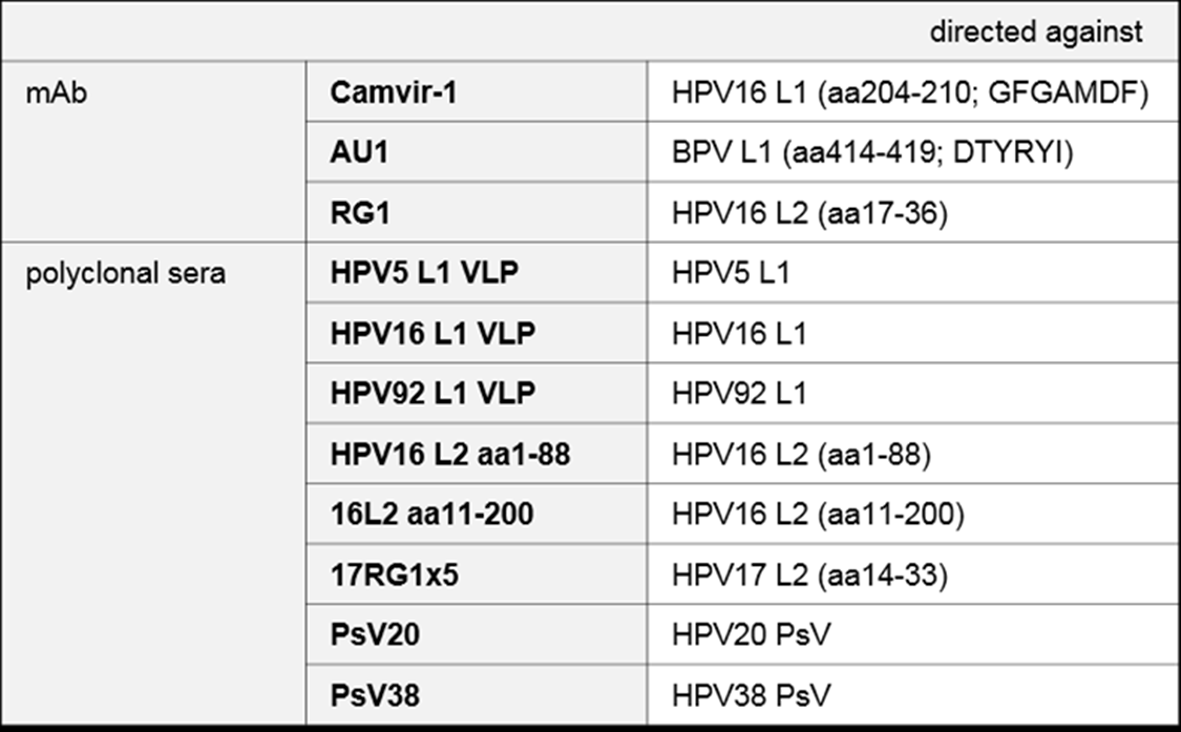

Supplement: S1 Table — (TIF) [file pone.0169533.s004.tif]

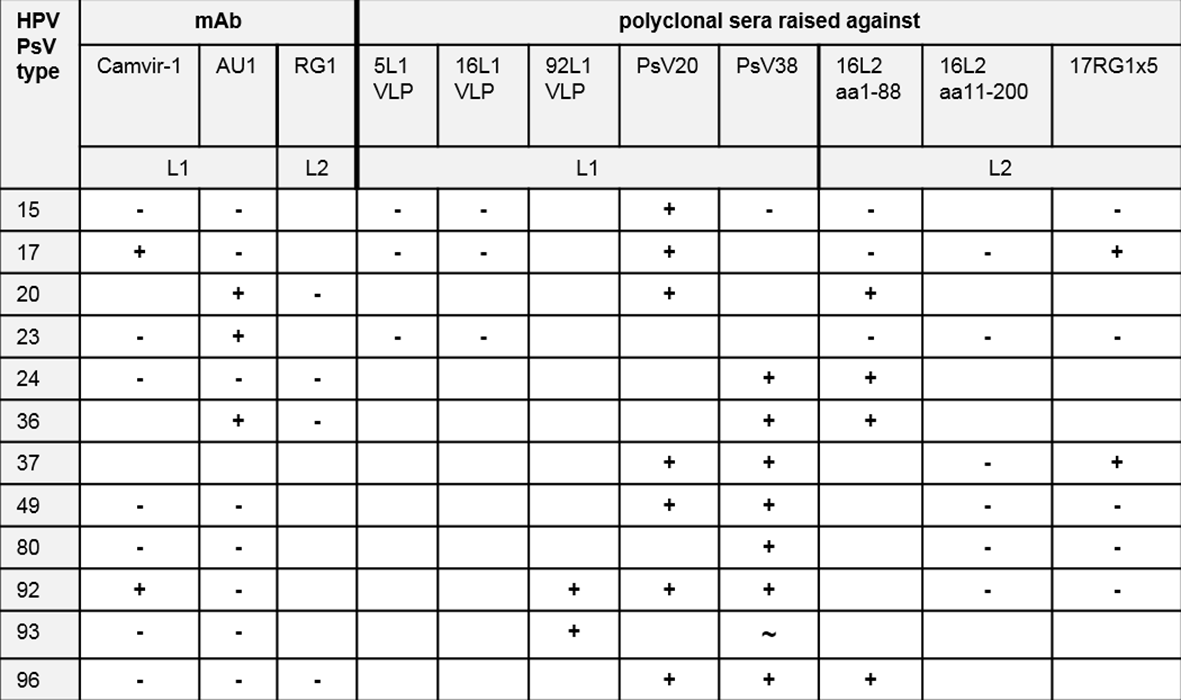

Supplement: S2 Table — MAb Camvir-1 or AU1, or polyclonal sera raised against the indicated L1 and/or L2 antigens, were used to detect L1 and L2 expression of a panel of beta HPV PsV by Western blot. Results are summarized. (-), (~) and (+) indicate negative, weak, or positive immunoreactivity, respectively. Empty boxes indicate that no experiment was done. (TIF) [file pone.0169533.s005.tif]

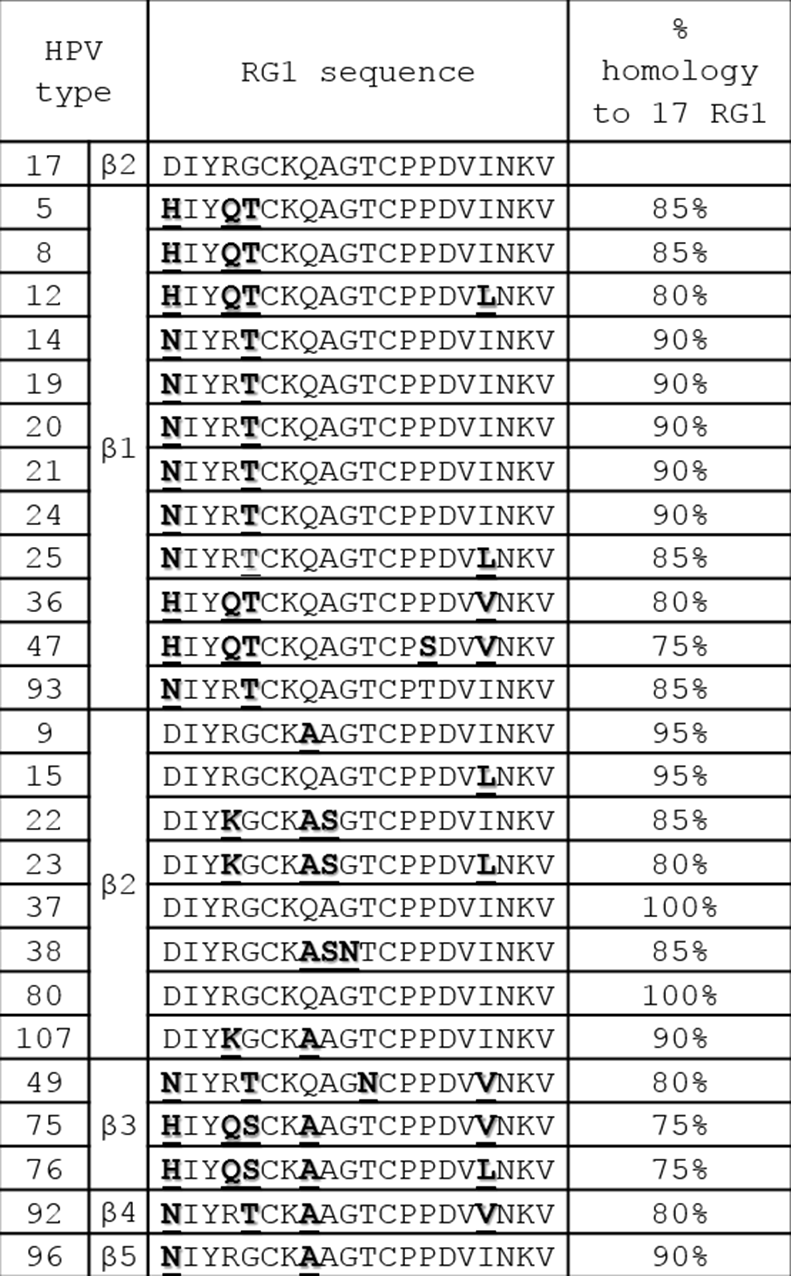

Supplement: S3 Table — (TIF) [file pone.0169533.s006.tif]

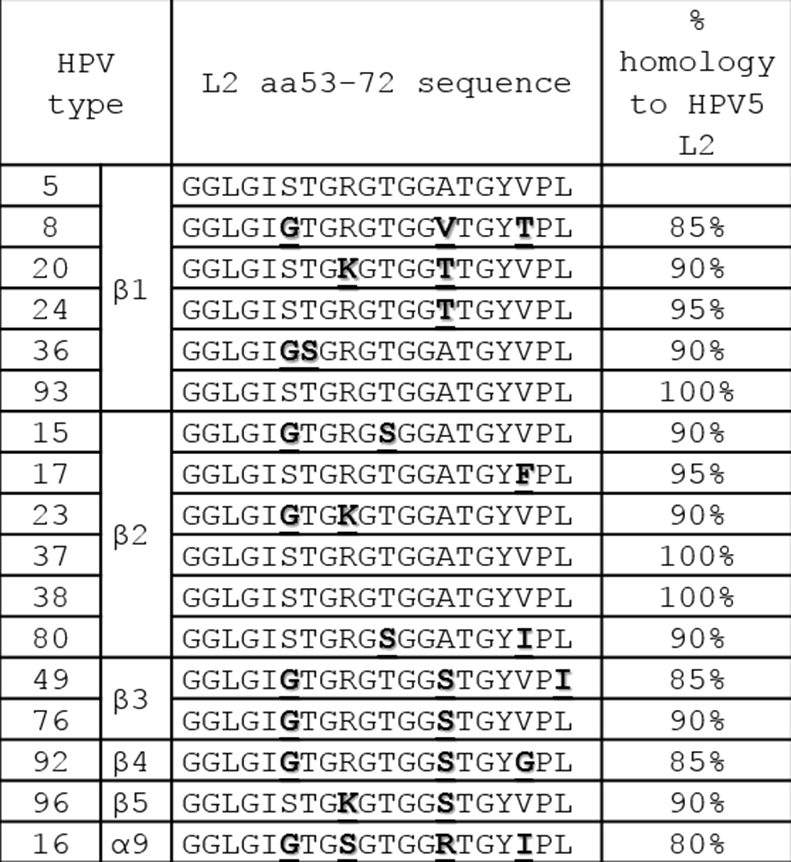

Supplement: S4 Table — (TIF) [file pone.0169533.s007.tif]

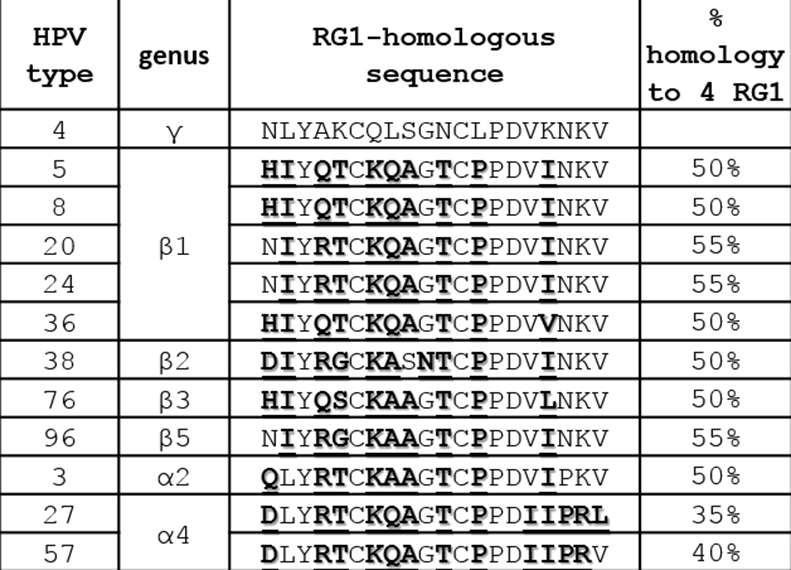

Supplement: S5 Table — (TIF) [file pone.0169533.s008.tif]
